# Tumor budding outperforms ypT and ypN classification in predicting outcome of rectal cancer after neoadjuvant chemoradiotherapy

**DOI:** 10.1186/s12885-019-6261-5

**Published:** 2019-11-01

**Authors:** Iryna Trotsyuk, Halina Sparschuh, Alice Josephine Müller, Konrad Neumann, Martin Kruschewski, David Horst, Sefer Elezkurtaj

**Affiliations:** 1 Institut für Pathologie, Charité – Universitätsmedizin Berlin, Campus Charité Mitte, Charitéplatz 1, 10117 Berlin, Germany; 2 Institut für Biometrie und Klinische Epidemiologie, Charité – Universitätsmedizin Berlin, Charitéplatz 1, 10117 Berlin, Germany; 3 Berlin Institute of Health (BIH), Anna-Louisa-Karsch 2, 10178 Berlin, Germany; 4 Klinik für Allgemein- und Viszeralchirurgie Klinikum, Müllroser Chaussee 7, 15236 Frankfurt (Oder), Germany

**Keywords:** Rectal cancer, Neoadjuvant therapy, Tumor budding, Prognostic factor

## Abstract

**Background:**

Budding is a complementary prognostic factor for colorectal cancer. In this study, we aimed to clarify the role of tumor budding in rectal cancer patients after preoperative chemoradiotherapy.

**Methods:**

A total of 124 patients with rectal cancer treated with neoadjuvant chemoradiotherapy and consecutive surgery were included. Surgical specimens were evaluated for budding and routine clinicopathological features. Budding was evaluated on hematoxylin and eosin (H&E)-stained slides and by cytokeratin immunohistochemical (IHC) staining.

**Results:**

A budding rate of 36.9% (*n* = 38) by H&E and 55.6% (*n* = 55) by IHC was observed. Budding was significantly associated with a high ypT and ypN status, poor differentiation, and low degrees of tumor regression. Moreover, budding was strongly predictive of a worse patient outcome, as measured by tumor recurrence or death. In multivariate analyses, budding remained the only significant parameter for overall survival and was even superior to the ypT and ypN status (budding in H&E: hazard ratio (HR) 2.72, 95% confidence interval (95% CI) 1.15–6.44, *p* = 0.023; budding in IHC: HR 5.19, 95% CI 1.62–16.61, *p* = 0.006).

**Conclusion:**

Budding is a strong prognostic predictor of survival in rectal cancer patients after neoadjuvant therapy. A standardized evaluation of tumor budding after neoadjuvant therapy may thus aid in risk stratification and guide the clinical management of patients with rectal cancer. Immunostaining can help to enhance the diagnostic accuracy and prognostic significance.

## Background

Locally advanced rectal cancers are treated with preoperative local radiation and simultaneous chemotherapy. Since the implementation of this therapy, the risk for local recurrence has notably decreased, and sphincter-preserving surgery is more often performed [1, 2]. After such intensive therapy, the initial morphology of the tumor is subject to considerable changes. Nevertheless, the evaluation of these histologically changed cancers remains the same as for tumors without neoadjuvant therapy. The tumor, node, metastasis (TNM) staging system, which is continuously updated, is widely used to predict outcomes and aids in clinical decision making in colorectal cancer. However, after preoperative therapy, the prognostic impact of the classical TNM system is subject to certain limitations, especially in tumors with wide fibrotic areas, i.e., a major therapeutic response [3]. Furthermore, the assessment of tumor regression, the relation of residual cancer cells to fibrosis, is an important feature of pathohistological evaluation protocols of rectal cancer after neoadjuvant therapy. Other morphological parameters have not yet been clarified as to whether they play a decisive role in the prognostic prediction of treated rectal cancer. They might constitute, however, a valuable addition to the TNM system and regression grading of treated rectal cancers.

An example of such a morphological parameter, the role of which is not yet clear in tumors treated with neoadjuvant therapy, is tumor budding. In rectal cancers without preoperative multimodality treatment, budding is associated with lymphovascular invasion [4–6], metastatic lymph nodes [4, 6–8], a higher TNM stage [6, 8], and distant metastasis [6, 9]. In colorectal carcinoma, budding is a strong adverse prognostic marker [4–9].

The aim of this retrospective study was to investigate whether tumor budding is a prognostic factor for survival in patients with rectal cancer who received neoadjuvant chemoradiotherapy. Special attention was paid to tumor budding assessed with the method introduced by Ueno et al. [10].

## Methods

### Patients

The study cohort included 124 consecutive patients with a biopsy-proven diagnosis of rectal adenocarcinoma and received radical surgery after neoadjuvant treatment between 2002 and 2011. All patients provided written consent to further investigate their tissue samples as well as the anonymous use of their clinical data. Patients under the age of 18 were not included in the study. Investigations on archived tissue and anonymized data were approved by the institutional ethics board (№. EA1/370/16). Primary clinical parameters and survival data were obtained from electronic health records. Distant metastasis had been excluded or detected at diagnosis and during the follow-up by abdominal ultrasound and chest radiography according to national guidelines. In cases of suspicion or ambiguity, computed tomography (CT) scans were performed [11]. Missing survival data at follow-up were gathered by delivering a questionnaire to the primary care physicians of the patients.

### Neoadjuvant therapy and surgery

All 124 included patients received long-course neoadjuvant therapy. Eighty patients were treated in strict compliance with the standard regimen, defined by a cumulated radiation dose of 50.4 Gy applied in 5 weekly fractions of 1.8 Gy using 18 MeV photons. These patients received a continuous infusion of 225 mg 5-FU per day and square meter of body surface for the duration of radiotherapy. Most of the remaining 44 patients received only slightly variant chemotherapy along with hyperfractionated radiation. After an interval of 4–6 weeks, total mesorectal (TME) surgery was performed.

### Pathological assessment

The quality of the total mesorectal excision was assessed using the Quirke criteria. Quirke grade 1 (poor) corresponds to irregular mesorectal fascia, with defects or incisions up to 1 square cm to the muscularis propria, irregular circumferential resection margin with small amount of mesorectal fat and low anterior safety margin. Quirke grade 2 (suboptimal) means that there is a moderate amount of mesorectum with some irregularity; moderate distal coning may be present. Quirke grade 3 (optimal) indicates that there is a good amount of mesorectum, a smooth surface, a good safety distance at the frontside and no defects in the mesorectum. Tissue sections were prepared from paraffin-embedded samples, mounted onto glass slides, stained with hematoxylin–eosin according to standard procedures, and examined with a Nikon ECLIPSE E200 microscope and a × 10 ocular lens. The pathological T and N stage (ypT and ypN, respectively) were evaluated according to the 7th AJCC TNM classification. The tumor regression grade of the resected tumor was assessed using the original score proposed by Dworak et al. [12]. Tumor regression was described as follows: Grade 0: no regression; Grade 1: dominant tumor mass with obvious fibrosis and/or vasculopathy; Grade 2: dominant fibrotic changes with few tumor cells or groups (easy to find); Grade 3: very few (difficult to find microscopically) tumor cells in fibrotic tissue; and Grade 4: no tumor cells, only a fibrotic mass (total regression or response). All formerly determined histopathological features, such as regression grade or pathological T and N stage, were retrospectively reevaluated by one trained observer (I.T.) who was blinded to patient outcomes and reviewed by a specialist gastrointestinal pathologist (S.E.).

### Tumor budding

Tumor budding was defined as a single tumor cell or a cluster of up to four tumor cells in the invasive front of the tumor or within the tumor. For quantifications, the sections were first scanned at a low power, and an area with maximal budding was identified. Then, tumor buds were counted in one field measuring 0.785 mm^2^ using a 20x objective lens. A field with five or more buds was viewed as budding positive (BD-1), while a field with four or fewer buds was viewed as budding negative (BD-0) [10].

### Immunohistochemical staining

In addition, to better understand tumor budding after neoadjuvant treatment and to highlight buds in detail, we also performed immunohistochemical (IHC) staining. There were 99 corresponding unstained tissue slides available for immunohistochemistry with a pan-cytokeratin antibody AE1/AE3. Staining was performed according to standard protocols provided by the automated Ventana BenchMark XT immunostainer (Ventana Medical Systems, Inc., Tucson, AZ, USA). Briefly, the tissue sections were deparaffinized and rehydrated and subjected to heat-induced epitope retrieval and endogenous peroxidase blocking with H_2_O_2_. Subsequently, the slides were incubated with a primary pan-cytokeratin antibody (clone AE1/AE3, dilution 1:500, DAKO) for 60 min and then with a horseradish peroxidase (HRP)-conjugated secondary antibody (DISCOVERY Universal Secondary Antibody (RUO)) for 32 min. This was followed by applying the chromogen 3,3′-diaminobenzidine-tetrahydrochloride (DAB) for 8 min and counterstaining with hematoxylin and bluing reagent (Ventana Medical Systems, Inc.) for 12 min. Budding was evaluated on IHC-stained slides with the same method and cut-off as described above for H&E-stained slides.

### Statistical analysis

We compared the BD-0 and BD-1 groups using the chi-squared test or Fisher’s exact test, as appropriate. Comparisons of the means of metrical variables, such as age, BMI, tumor size, circumferential resection margin between the BD-0 and BD-1 groups were performed using a t-test for independent samples. Univariate and multivariate Cox proportional hazards regression models were used to estimate hazard ratios (HRs) with 95% confidence intervals (95% CIs). For categorical variables, the lowest value served as the reference category. The categorical variables used in the univariate analyses were gender (male versus female), American Society of Anesthesiologists (ASA) classification (2 and 3 versus 1), cM stage, type of resection (Abdominoperineal resection versus lower anterior resection), adjuvant therapy, Quirke Grade (poor versus moderate and good), higher ypT stage (ypT3–4 versus ypT0–2), positive ypN stage (ypN+ versus ypN0), higher histological and regressive grading, vascular and perineural invasion (V1, L1, Pn1), quality of resection (R+ versus R0) and positive budding. The continuous variables used in univariate analyses were age at surgery, body mass index (BMI), tumor size and circumferential resection margin (CRM). Covariates and factors included in the multivariate regression analysis were budding and ypT and ypN stage. Dichotomization of ypT and ypN stages was used to avoid overfitting of the model. The primary endpoints of the study were the hazard ratios for disease-free survival (DFS) and overall survival (OS). Overall survival (OS) was defined as the time from the date of surgery to the date of death from any cause. Disease-free survival (DFS) was defined as the time from the date of surgery to the date of pelvic recurrence and/or distant disease or death from any cause. Kaplan-Meier survival curves show the influence of tumor budding on survival. The different budding categories (BD-0 and BD-1) in the plotted DFS and OS curves were compared using the log-rank test. With the kappa value, we evaluated the consistency of H&E-stained slides with IHC-stained slides. All tests were two-sided, and the level of significance was set at α = 0.05. We performed all statistical analyses using SPSS Statistics 24.0 Software (SPSS, Inc., Chicago, IL).

## Results

The initial study cohort included 124 patients, 87 men (70.2%) and 37 women (29.8%), with a mean age of 64.7 years (range 34–87 years). The TNM classification before neoadjuvant treatment was composed as follows: cT2 in 12 patients (9.7%), cT3 in 92 patients (74.2%) and cT4 in 20 patients (16.1%); cN0 in 14 patients (11.3%) and cN+ in 110 patients (88.7%); cM0 in 111 (89.5%) and cM1 in 13 (10.5%) patients. Lower anterior resection (LAR) was performed on 92 patients (74.2%), abdominal perineal resection on 32 patients (25.8%). The average number of harvested lymph nodes was 18 (range 5–67). The quality of mesorectum specimens was poor in 5 (4.0%), suboptimal in 19 (15.3%) and optimal in 86 (69.4%) resected rectal cancers. In 14 (11.3%) it was not described. A complete resection with R0 status was achieved in 118 patients (95.2%). Adjuvant chemotherapy was received in 71 (57.3%) cases, while in 32 (25.8%) cases the tumor board decided against adjuvant therapy. For 21 (16.9%) patients information on adjuvant therapy was not available.

There were seven local recurrences (5.6%) and eighteen distant recurrences (14.5%) in the follow-up period. Thirty-two patients (25.8%) died during the follow-up period. The mean time for recurrence was 28.4 months (standard deviation 24.6 months, maximum 94 months). The mean follow-up time was 54.7 months (standard deviation 35.5 months, maximum 134 months).

### Evaluation of tumor budding by H&E and IHC

**Fig. 1:**
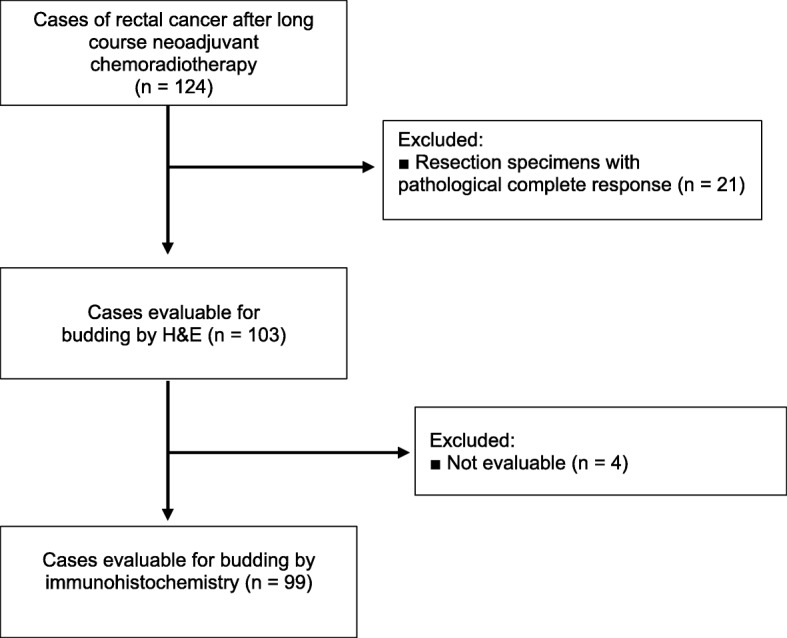
Flow chart of histological analysis for the study cohort

**Fig. 2:**
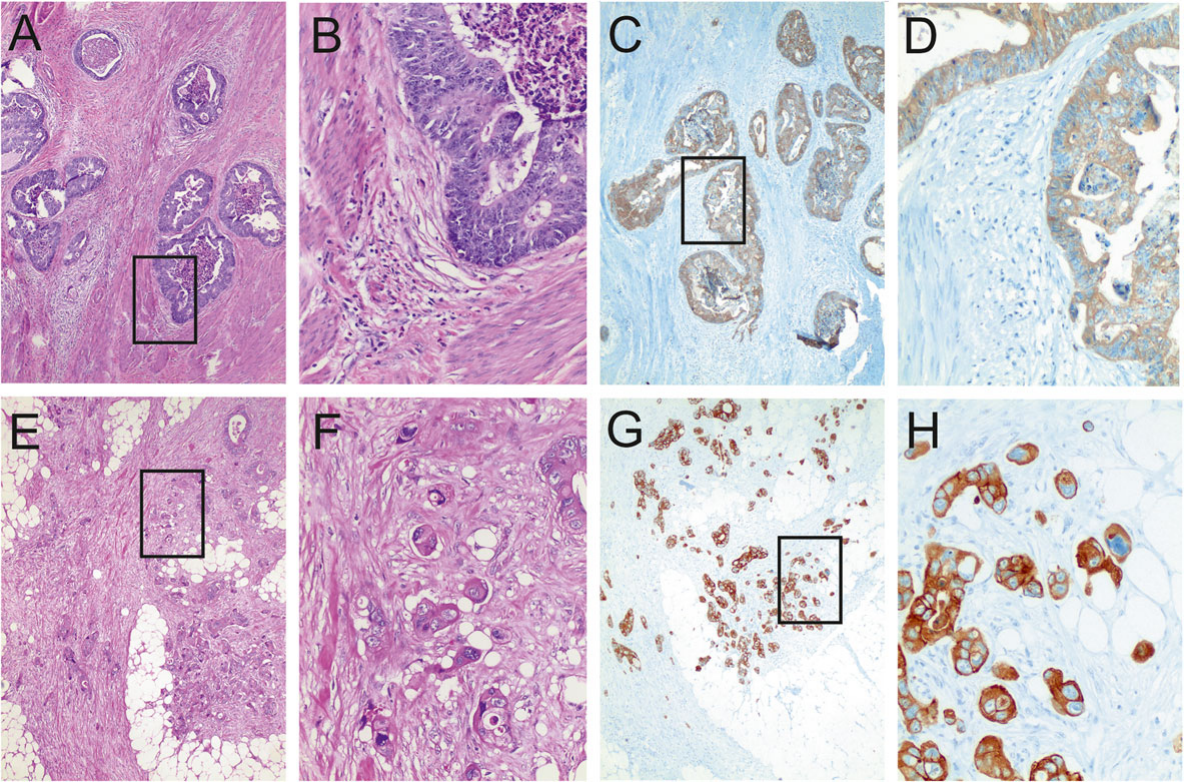
Comparison of tumor budding in neoadjuvant treated rectal cancers in both hematoxylin-eosin and in immunohistochemical staining. Tumor budding was defined as a single tumor cell or a cluster up to four tumor cells at the invasive front or within the tumor as well. Tumor buds were counted in one field measuring 0.785 mm^2^ using a 20x objective lens. A field with 4 buds or fewer was viewed as budding negative (**a-d**), a field with 5 or more buds was viewed as budding positive (**e-h**). Boxed areas are shown in a higher magnification on the right side of the corresponding picture. (Original magnification and staining method: (**a**) × 40, H&E; (**b**) × 200, H&E; (**c**) × 40, AE1/AE3; (**d**) × 200, AE1/AE3; (**e**) × 40, H&E; (**f**) × 200, H&E; (**g**) × 40, AE1/AE3; (**h**) × 200, AE1/AE3;)

In the following tumor budding analyses, we examined only specimens with residual tumor. Twenty-one specimens with pathological complete response (pCR) were excluded from further statistical analyses. Without 21 pCR cases, there were 103 cases available for analysis of H&E-stained tissue sections, and 99 cases for analysis of IHC-stained tissue sections (Fig. 1). The examination of H&E-stained sections showed 38 (36.9%) budding-positive cases and 65 (63.1%) budding-negative cases. On IHC-stained sections, 44 cases (44.4%) were budding negative, and 55 cases (55.6%) were budding positive; thus, there was a higher percentage compared to H&E-stained slides. Figure 2 illustrates an example of a budding-negative case and a budding-positive case from both staining methods.

To evaluate the consistency of budding evaluated on H&E- or IHC-stained sections, we used fourfold tables (Table 1). After immunohistochemical staining, there were more budding-positive cases. The assessment of tumor budding on H&E and IHC reached good agreement, with a kappa value of 0.609. The evaluation of budding on H&E-stained slides compared well with the evaluation of budding on IHC-stained slides.

**Table 1:** Comparison of cases where budding was evaluated on H&E with cases budding evaluated on IHC

		Budding on IHC		
		negative	positive	n
Budding on H&E	**negative**	44	20	64
	**positive**	0	35	35
	**n**	44	55	99

### Tumor budding is associated with adverse clinicopathological features

As analyzed by H&E staining, budding was found more frequently in specimens of elderly patients (*p* = 0.032), in patients with a lower BMI (*p* = 0.042) and in patients with a higher ASA score (*p* = 0.022). Patients in whom lower anterior resection was performed had significantly less budding (*p* = 0.004). Budding was also significantly associated with a higher T stage, both before and after neoadjuvant therapy (for cT stage *p* = 0.005 and for ypT stage *p* = 0.001), metastatic lymph nodes (for ypN stage *p* = 0.006), a poorer level of histological differentiation (*p* = 0.021), a lower response according to Dworak’s tumor regression grading scale (*p* = 0.001), venous or perineural invasion (*p* = 0.030 and *p* = 0.001), and a worse outcome in subsequent observations, such as cancer recurrence (*p* = 0.002) or death (*p* = 0.006). Tumors with a smaller circumferential resection margin and a poor quality of mesorectal excision (Quirke Grade poor) also had significantly more budding (*p* = 0.010 and *p* = 0.004, respectively). There were no significant associations between budding and sex (*p* = 0.501), the mean macroscopically evaluated tumor size (*p* = 0.192), synchronous metastasis at the time of surgery (*p* = 0.066), or metastatic lymph nodes before neoadjuvant treatment (*p* = 0.742). The parameters mentioned above can be found in Tables 2 and 3. The clinicopathological features of budding in IHC-stained specimens are listed in Tables 2 and 3.

**Table 2:** Tumor budding and associations with clinical features

	Budding on H&E					Budding on IHC				
	negative		positive		*P*	negative		positive		*P*
All patients, n (%)	65	(63.1)	38	(36.9)		44	(44.4)	55	(55.6)	
Age										
Mean, years (SD)	62.5	(9.2)	67.1	(10.8)	**0.032**	61.8	(9.8)	65.7	(9.8)	0.055
Sex, n (%)										
Male	48	(65.8)	25	(34.2)	0.501	34	(47.9)	37	(52.1)	0.370
Female	17	(56.7)	13	(43.3)		10	(35.7)	18	(64.3)	
BMI										
Mean, kg/m^2^ (SD)	25.8	(3.9)	23.6	(5.7)	**0.042**	25.6	(3.2)	24.6	(5.6)	0.252
ASA classification, n (%)										
1	12	(85.7)	2	(14.3)	**0.022**	9	(64.3)	5	(35.7)	0.096
2	47	(64.4)	26	(35.6)		31	(44.9)	38	(55.1)	
3	6	(37.5)	10	(62.5)		4	(25.0)	12	(75.0)	
cT stage, n (%)										
T0–1	–	–	–	–	**0.005**	–	–	–	–	**0.011**
T2	5	(55.6)	4	(44.4)		2	(22.2)	7	(77.8)	
T3	54	(72.0)	21	(28.0)		39	(53.4)	34	(46.6)	
T4	6	(31.6)	13	(68.4)		3	(17.6)	14	(82.4)	
cN stage, n (%)										
N0	7	(70.0)	3	(30.0)	0.742	7	(70.0)	3	(30.0)	0.104
N+	58	(62.4)	35	(37.6)		37	(41.6)	52	(58.4)	
cM stage, n (%)										
M0	60	(66.7)	30	(33.3)	0.066	42	(47.2)	47	(52.8)	0.178
M1	5	(38.5)	8	(61.5)		2	(20.0)	8	(80.0)	
Type of resection, n (%)										
LAR	53	(71.6)	21	(28.4)	**0.004**	39	(53.4)	34	(46.6)	**0.003**
APR	12	(41.4)	17	(58.6)		5	(19.2)	21	(80.8)	
Adjuvant therapy, n (%)										
Yes	42	(66.7)	21	(33.3)	0.218	27	(45.0)	33	(55.0)	0.741
No	12	(52.2)	11	(47.8)		9	(40.9)	13	(59.1)	
*Not described*	*11*		*6*			*8*		*9*		
Relapse in the follow-up period, n (%)										
No	57	(71.3)	23	(28.7)	**0.002**	41	(53.9)	35	(46.1)	**0.001**
Yes	8	(34.8)	15	(65.2)		3	(13.0)	20	(87.0)	
Type of relapse, n (%)										
Local recurrence	2	(50.0)	2	(50.0)	**0.015**	1	(25.0)	3	(75.0)	**0.003**
Liver	2	(28.6)	5	(71.4)		0	(0)	7	(100)	
Lung	3	(33.3)	6	(66.7)		1	(11.1)	8	(88.9)	
Cerebral	1	(33.3)	2	(66.7)		1	(33.3)	2	(66.7)	
Survival status in follow-up, n (%)										
Alive	54	(71.1)	22	(28.9)	**0.006**	40	(53.3)	35	(46.7)	**0.002**
Dead	11	(40.7)	16	(59.3)		4	(16.7)	20	(83.3)	

Abbreviations: *pCR* pathological complete response, *P* = *P*-value, *SD* standard deviation, *BMI* body mass index, *ASA* American Society of Anesthesiologists, *LAR* low anterior resection, *APR* abdominoperineal excision. Significant *p*-values are represented in bold type

**Table 3:** Tumor budding and associations with pathological features

	Budding on H&E					Budding on IHC				
	negative		positive		*P*	negative		positive		*P*
All patients, n (%)	65	(63.1)	38	(36.9)		44	(44.4)	55	(55.6)	
Tumor size										
Mean, cm (SD)	2.9	(1.4)	3.4	(2.2)	0.192	2.7	(1.4)	3.3	(2.0)	0.064
CRM										
Mean, mm (SD)	18.4	(18.3)	9.4	(15.8)	**0.010**	19.1	(15.6)	12.4	(19.3)	0.061
Quirke Grade, n (%)										
Poor	0	(0)	5	(100)	**0.004**	0	(0)	4	(100)	0.218
Suboptimal	13	(81.3)	3	(18.8)		8	(50.0)	8	(50.0)	
Optimal	49	(69.0)	22	(31.0)		34	(48.6)	36	(51.4)	
*Not described*	*3*		*8*			*2*		*7*		
ypT stage, n (%)										
T0–1	10	(100)	0	(0)	**0.001**	9	(90.0)	1	(10.0)	**0.001**
T2	26	(81.3)	6	(18.8)		19	(61.3)	12	(38.7)	
T3	25	(49.0)	26	(51.0)		15	(30.6)	34	(69.4)	
T4	4	(40.0)	6	(60.0)		1	(11.1)	8	(88.9)	
ypN stage, n (%)										
N0	40	(76.9)	12	(23.1)	**0.006**	29	(58.0)	21	(42.0)	**0.021**
N1	20	(54.1)	17	(45.9)		12	(32.4)	25	(67.6)	
N2	5	(35.7)	9	(64.3)		3	(25.0)	9	(75.0)	
Grading, n (%)										
G1	2	(66.7)	1	(33.3)	**0.021**	2	(66.7)	1	(33.3)	0.290
G2	47	(67.1)	23	(32.9)		31	(44.9)	38	(55.1)	
G3	16	(64.0)	9	(36.0)		11	(47.8)	12	(52.2)	
G4	0	(0)	5	(100)		0	(0)	4	(100)	
Dworak’s regression, n (%)										
Grade 1	2	(20.0)	8	(80.0)	**0.001**	1	(10.0)	9	(90.0)	**0.001**
Grade 2	47	(62.7)	28	(37.3)		29	(40.8)	42	(59.2)	
Grade 3	16	(88.9)	2	(11.1)		14	(77.8)	4	(22.2)	
Venous invasion, n (%)										
V0	58	(68.2)	27	(31.8)	**0.030**	38	(46.3)	44	(53.7)	0.404
V1	7	(38.9)	11	(61.1)		6	(35.3)	11	(64.7)	
Lymphatic invasion, n (%)										
L0	61	(66.3)	31	(33.7)	0.055	44	(50.0)	44	(50.0)	**0.001**
L1	4	(36.4)	4	(63.6)		0	(0)	11	(100)	
Perineural invasion, n (%)										
Pn0	62	(71.3)	25	(28.7)	**0.001**	43	(51.2)	41	(48.8)	**0.001**
Pn1	3	(18.8)	13	(81.3)		1	(6.7)	14	(93.3)	
Resection margin, n (%)										
R0	64	(66.0)	33	(34.0)	**0.021**	43	(45.7)	51	(54.3)	0.792
R1	1	(20.0)	4	(80.0)		1	(25.0)	3	(75.0)	
R2	0	(0)	1	(100)		0	(0)	1	(100)	

Abbreviations: *pCR* pathological complete response, *P P*-value, *SD* standard deviation, *CRM* circumferential resection margin. Significant *p*-values are represented in bold type

### Many factors influence the survival of rectal cancer patients

In univariate analysis, factors such as a higher ypT stage, metastatic lymph nodes, vascular and perineural invasion, synchronous metastasis at the time of surgery, poorly differentiated and macroscopically larger tumors and positive budding (evaluated in both staining methods) had a significantly negative impact on disease-free survival. A higher BMI, larger circumferential resection margins on tumor specimens and more regression had a significantly positive impact on disease-free survival. All these variables with the corresponding hazard ratios can be found in Table 4.

**Table 4:** Univariate cox regression analysis of DFS

	Disease-free survival		
Parameter	HR	95% CI	*P*
Age	1.03	[0.99; 1.06]	0.124
Male vs. female	0.63	[0.32; 1.24]	0.177
BMI	**0.91**	[0.85; 0.98]	**0.009**
ASA 2 and 3	2.04	[0.62; 6.66]	0.238
cM1	**7.58**	[3.42; 16.79]	**< 0.001**
Type of resection: APR vs. LAR	1.83	[0.92; 3.62]	0.083
Adjuvant therapy recieved	0.99	[0.44; 2.21]	0.979
Tumor size	**1.02**	[1.00; 1.04]	**0.048**
CRM	**0.93**	[0.88; 0.97]	**0.002**
Poor Quirke Grade	1.78	[0.99; 3.18]	0.053
Higher ypT stage [ypT3–4]	**4.20**	[1.83; 9.66]	**0.001**
ypN+	**2.34**	[1.16; 4.68]	**0.017**
Histological Grading	**1.97**	[1.20; 3.25]	**0.008**
Tumor regression Grading	**0.35**	[0.16; 0.75]	**0.007**
Budding positive H&E	**3.54**	[1.82; 6.89]	**< 0.001**
Budding positive IHC	**6.23**	[2.57; 15.31]	**< 0.001**
V1	**2.41**	[1.16; 5.03]	**0.019**
L1	**2.75**	[1.20; 6.32]	**0.017**
Pn1	**5.49**	[2.65; 11.37]	**0.001**
R+	1.51	[0.36; 6.35]	0.573

Abbreviations: *DFS* disease-free survival, *HR* hazard ratio, *95% CI* 95% confidence interval, *P* P-value, *BMI* body mass index, *ASA* American Society of Anesthesiologists, *APR* abdominoperineal excision, *LAR* low anterior resection, *CRM* circumferential resection margin, *Pn1* perineural invasion; R+ = invaded margin. Significant *p*-values and corresponding hazard ratios are represented in bold type

In univariate analyses on overall survival, age and synchronous metastasis at the time of surgery, a poorer quality mesorectal excision, a higher ypT stage, perineural invasion, poorly differentiated tumors and positive budding, independent of the staining method, had a significantly negative impact on overall survival. Lymph node stage had no significant influence on overall survival. Similar to disease-free survival, prolonged overall survival was associated with tumor specimens with larger circumferential resection margins and patients with more regression and in patients with a higher BMI. All these variables with the corresponding hazard ratios can be found in Table 5.

**Table 5:** Univariate cox regression analysis of OS

	Overall survival		
Parameter	HR	95% CI	*P*
Age	**1.06**	[1.01; 1.10]	**0.010**
Male vs. female	0.54	[0.25; 1.16]	0.114
BMI	**0.97**	[0.84; 0;97]	**0.006**
ASA 2 and 3	2.21	[0.52; 9.37]	0.282
cM1	**6.53**	[2.74; 15.54]	**0.001**
Type of resection: APR vs. LAR	1.77	[0.80; 3.89]	0.156
Adjuvant therapy	1.23	[0.45; 3.37]	0.681
Tumor size	1.01	[0.98; 1.03]	0.554
CRM	**0.95**	[0.90; 0.99]	**0.043**
Poor Quirke Grade	**2.72**	[1.47; 2.47]	**0.001**
Higher ypT stage [ypT3–4]	**3.01**	[1.20; 7.55]	**0.019**
ypN+	1.64	[0.74; 3.63]	0.220
Histological Grading	**2.24**	[1.26; 3.97]	**0.006**
Tumor regression Grading	**0.19**	[0.07; 0.51]	**0.001**
Budding positive H&E	**3.43**	[1.57; 7.52]	**0.002**
Budding positive IHC	**5.76**	[1.95; 17.01]	**0.002**
V1	1.60	[0.64; 3.99]	0.316
L1	1.90	[0.70; 5.18]	0.209
Pn1	**3.76**	[1.60; 8.85]	**0.002**
R+	1.01	[0.14; 7.49]	0.993

Abbreviations: *OS* overall survival, *HR* hazard ratio, *95% CI* 95% confidence interval, *P* P-value, *BMI* body mass index, *ASA* American Society of Anesthesiologists, *APR* abdominoperineal excision, *LAR* low anterior resection, *CRM* circumferential resection margin, *Pn1* perineural invasion, R+ = invaded margin. Significant *p*-values and corresponding hazard ratios are represented in bold type

### Budding is an independent prognostic factor for disease-free survival and overall survival in multivariate cox proportional hazards regression models

In the multivariate analysis, budding scored on H&E-stained sections (HR 2.34, 95% CI 1.14–4.79; *p* = 0.020) and ypT stage (HR 2.85, 95% CI 1.16–7.02; p = 0.023) were both independent predictors of disease-free survival (Table 6). In the multivariate analysis, when budding was evaluated on IHC-stained sections, positive budding (HR 4.59, 95% CI 1.79–11.72; p = 0.001) remained the only independent prognostic factor (Table 7). In the multivariate regression analysis of overall survival, only tumor budding remained a significant parameter (H&E: HR 2.72, 95% CI 1.15–6.44, *p* = 0.023; IHC: HR 5.19, 95% CI 1.62–16.61, p = 0.006) and was even superior to the ypT and ypN status (Tables 8 and 9).

**Table 6:** Multivariate cox regression analysis of DFS: Budding evaluated on H&E

	Disease-free survival		
Parameter	HR	95% CI	*P*
Positive Budding H&E	**2.34**	[1.14; 4.79]	**0.020**
Higher ypT stage [ypT3–4]	**2.85**	[1.16; 7.02]	**0.023**
ypN+	1.34	[0.63; 2.83]	0.449

**Table 7:** Multivariate cox regression analysis of DFS: Budding evaluated on IHC

	Disease-free survival		
Parameter	HR	95% CI	*P*
Positive Budding IHC	**4.59**	[1.79; 11.72]	**0.001**
Higher ypT stage [ypT3–4]	2.16	[0.87; 5.34]	0.095
ypN+	1.29	[0.60; 2.77]	0.516

**Table 8:** Multivariate cox regression analysis of OS: Budding evaluated on H&E

	Overall survival		
Parameter	HR	95% CI	*P*
Positive Budding H&E	**2.72**	[1.15; 6.44]	**0.023**
Higher ypT stage [ypT3–4]	2.17	[0.78; 6.06]	0.140
ypN+	1.12	[0.46; 2.71]	0.803

**Table 9:** Multivariate cox regression analysis of OS: Budding evaluated on IHC

	Overall survival		
Parameter	HR	95% CI	***P***
Positive Budding IHC	**5.19**	[1.62; 16.61]	**0.006**
Higher ypT stage [ypT3–4]	1.50	[0.53; 4.26]	0.443
ypN+	1.18	[0.47; 2.92]	0.727

Abbreviations: *DFS* disease-free survival, *OS* overall survival, *HR* hazard ratio, *95% CI* 95% confidence interval, *P P*-value. Significant *p*-values and corresponding hazard ratios are represented in bold type

### The prognostic impact of budding is confirmed by Kaplan–Meier survival analysis

**Fig. 3:**
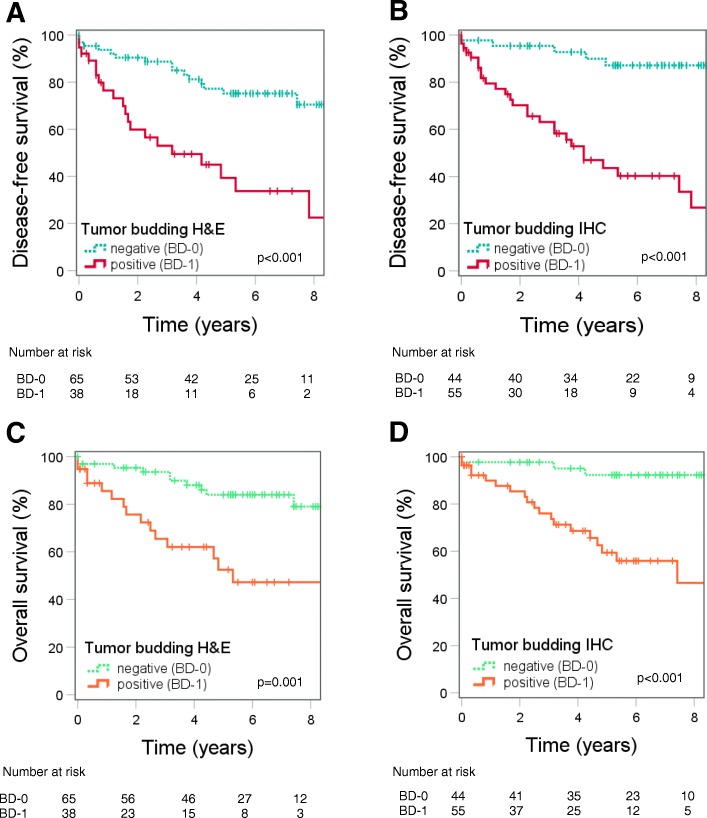
Kaplan-Meier curves for disease-free survival (DFS) and overall survival (OS). Independently of the staining method, DFS and OS were significant poorer on budding positive cases (BD-1). **a** DFS and budding evaluated on H&E (Log-rank test *p* < 0.001). **b** DFS and budding evaluated on IHC (Log-rank test *p* < 0.001). **c** OS and budding evaluated on H&E (Log-rank test *p* = 0.001). **d** OS and budding evaluated on IHC (Log-rank test *p* < 0.001)

With the H&E staining method, for patients with budding-positive tumors, the five-year disease-free survival rate was 39.0%, and for those without budding, the rate was 75.0%. With the IHC staining method, for patients with budding-positive tumors, the five-year disease-free survival rate was 44.0%, and for those without budding, the rate was 87.0%. Furthermore, for patients with positive budding evaluated on H&E-stained sections, the five-year overall survival rate was 53.0%, and for those without budding, the rate was 84.0%. On IHC-stained sections, the five-year overall survival rate was 59.0% for patients with budding-positive tumors and 92.0% for those without budding. Independent of the staining method, patients with positive budding had significantly poorer DFS and OS compared to those without budding (Fig. 3).

## Discussion

In the present study, we investigated whether tumor budding is a prognostic factor in patients with rectal adenocarcinoma treated with neoadjuvant therapy. Our results showed a strong connection between posttreatment budding and a more aggressive tumor biology, i.e., correlation with adverse clinicopathological features, such as deeper tumor infiltration or a higher frequency of lymph node metastases. Irrespective of the staining method used, patients with tumor budding had a significantly worse prognosis for disease-free survival and overall survival. These aspects have already been described in patients with chemotherapy-naïve colorectal cancer [4–9] and included as a recommendation in major national guidelines for the assessment of early invasive cancer [11, 13, 14].

Budding has been described as a prognostic feature after chemoradiotherapy in rectal cancer patients in several publications with the general limitation of a retrospective study design. In previous studies, budding was reported in 10.1–63.2% of cases due to different methodologies used for evaluation [3, 15–20]. Budding has been shown to be a negative prognostic factor for survival in different kinds of study designs and for a broad range of cut-offs. However, most of the previous studies could demonstrate effects on survival only in univariate analysis or limited to disease free survival [15–19]. Including patients with complete response in the analysis appeared to attenuate the prognostic impact of tumor budding. In our opinion, it is self-evident that budding cannot be evaluated in patients with a complete response. Therefore, in our study, we focused on cases with poor response in order to stratify the outcome of patients with residual tumor burden. By this approach, we were able to demonstrate a strong impact on disease free survival and overall survival in univariate and multivariate analysis.

Of the most recent studies, Jäger et al. [3] can be compared to our own study. As in our study, they evaluated budding not only at the invasive front but also throughout the tumor. The high budding rate of 63.2% compared to our results can be explained by the low cut-off of two buds in one microscopic field, whereas in our study a cutoff of 5 buds was used according to standard criteria of Ueno et al. [10]. As in our study, budding remained a significant parameter in multivariate analysis for disease free survival. However they failed to demonstrate this for overall survival, presumably, because patients with complete response were included in the statistical analysis.

Only one previous study claimed that a single cell pattern of growth in the invasive front was a prognostic factor for prolonged cancer-specific survival [21]. They interpreted the single-cell growth pattern as an indicator of tumor cell regression. However, they did not evaluate budding as a standardized parameter but rather as a semiquantitative score of the tumor growth pattern. Furthermore, patients with a complete pathological response were included in the survival analysis, undermining the impact of budding as a parameter for the stratification of patients with a poor response.

In our study, immunohistochemical staining showed that budding had a considerable prognostic influence and was even superior to that of conventional parameters such as ypT and ypN stage, which have been used in routine so far. Therefore, the assumption arises that the assessment of posttreatment budding may improve the commonly used TNM classification for stratifying rectal cancer patients treated with neoadjuvant therapy and for predicting prognosis.

However, there is still a general lack of a unified definition of tumor budding. At the International Tumor Budding Consensus Conference (ITBCC) in 2016, a consensus for a standardized definition of budding and for an evaluation method was reached, but only for colorectal cancers without neoadjuvant chemoradiotherapy so far [22]. Tumor budding was defined as a single cell or a cluster of up to four tumor cells assessed in one hotspot measuring 0.785 mm^2^ at the invasive front. Furthermore, a three-tier system was recommended with a whole budding count to allow adequate risk stratification. The documentation of tumor budding after neoadjuvant therapy has not yet been suggested for daily diagnostic practice because of data gaps in its prognostic value in treated rectal cancers as well as a lack of a standardized evaluation method for these cancers.

The initial morphology of the tumor is often modified after chemoradiation, with phenomena such as heavy fibrosis, breaking up of the glandular tumor structures and necrotic areas. Due to these factors, the assessment of budding proposed by the ITBCC becomes challenging. It should also be mentioned that after neoadjuvant therapy, tumor borders may appear fragmented, so the tumors occasionally form several invasive fronts in the context of fibrosis and inflammation. Owing to these histological changes, we assessed tumor budding not only at the utmost invasive front (such as in cancers without chemoradiation) but also in-between invasive foci. Lugli et al. [23] and Rieger et al. [24] showed that intratumoral budding in chemotherapy-naïve patients with colorectal cancer is generally associated with peritumoral budding. They found that as long as the observer investigates the densest region with budding, it does not matter whether buds are detected at the invasive front or within the tumor. Our method used to assess budding without being limited to the invasive front in neoadjuvant-treated cancers was fundamentally based on those findings. With our method, we were able to address the abovementioned problems occurring after preoperative therapy while keeping the method relatively simple and potentially reproducible for other observers.

For our budding analyses, we used the one hotspot method, as recommended by the ITBCC and originally proposed by Ueno et al. [10]. It is a fast and simple way to subdivide patients into two different categories that are prognostically highly relevant. Even in patients with little residual tumor after preoperative therapy, the method was able to find high-risk patients. Although the cut-off was set to merely 5 buds per hotspot, as proposed for the stratification of pT1 carcinomas in polyps [10], it was possible to apply the same cut-off for locally advanced cancers, with a resulting high impact of both disease-free survival and overall survival.

In addition to H&E staining, we used IHC staining to make buds more readily visible. Kai et al. [25] were able to show that IHC can reduce interobserver variability in the evaluation of budding between unskilled observers. This would make IHC suitable for training pathologists who are inexperienced in this field. As described in previous studies for colorectal cancers without prior chemotherapy, cytokeratin staining detected more budding-positive cases [26]. In our study, we detected more budding-positive cases by the means of IHC staining as well, and this method improved the prognostic value of tumor budding assessment. IHC helped to stratify patients into even more meaningful risk groups than H&E staining. When analyzed with IHC staining, fewer budding-negative cases were found, and these had a better prognosis than budding-negative cases found by H&E (five-year disease-free survival rate: 87% vs 75%; and five-year overall survival rate: 92% vs 84%). The employed cut-off may therefore identify patients with a favorable prognosis who might be able to refrain from adjuvant therapy. If the cut-off for budding on IHC was higher, high-risk patients would more likely come to light. In these patients, more intensive aftercare might be recommended. The ideal cut-off for the evaluation of budding in IHC-stained sections still needs to be investigated. In tumors without neoadjuvant therapy, the ITBCC recommends the use of IHC in difficult cases (such as for distinguishing buds from peritumoral inflammation reaction), but the final evaluation should still be performed on H&E-stained slides [22]. In the case of pretreated tumors, the role of IHC and H&E staining still needs to be determined. Since, for example, more tumor inflammation occurs in such cases, IHC might play a more central role in the evaluation of these tumors. So far, only two previous studies regarding IHC staining in posttreatment budding exist [17, 18]. Concerning this issue, more investigations should be performed.

The main limitation of this work is its retrospective nature. It also needs to be determined whether the number and composition of included patients may vary among patients with different ethnic backgrounds. In addition, the recorded number of events during the follow-up could be a limitation as well, especially in the interpretation of the Cox regression models due to the possible lack of outcome data. Nevertheless, current data on the prognosis of tumor budding in rectal cancers treated with neoadjuvant therapy are unsatisfactory.

It is remarkable that lymph node status did not have a significant effect on outcome in our multivariate survival analysis. In fact, previous studies of neoadjuvant therapy in rectal cancer found strong associations of lymph node status with survival [27, 28]. However, in these studies, patients with complete pathological response were included in the analysis, who were here excluded due to being non-informative for tumor budding. We speculate that the specific case selection in our study may be the cause of such discrepancies.

## Conclusion

In conclusion, our study provides further information to understand and evaluate budding in rectal carcinomas treated with neoadjuvant therapy while analyzing budding on H&E- and immunostained specimens. Immunohistochemical staining can substantially enhance the diagnostic accuracy and prognostic impact. Tumor budding should be taken more seriously into account in daily diagnostic practice, since it represents an additional and independent prognostic factor for therapeutic decision making, even in rectal cancer patients after preoperative therapy. The method of Ueno is well suited for this purpose because of its fast and simple method of evaluating budding-positive cases. However, more knowledge about budding and a consensus about the best method used to assess buds after perioperative therapy are strongly needed to standardize the process and to speed up its application in pathological protocols for treated rectal cancers.

## Data Availability

The datasets used and/or analyzed during the current study are available from the corresponding author on reasonable request.

## Abbreviations

5-FU: 5-fluorouracil
95% CI: 95% confidence interval
APR: Abdominoperineal excision
ASA: American Society of Anesthesiologists
BD-0: Budding negative
BD-1: Budding positive
BMI: Body mass index
CRM: Circumferential resection margin;
CT: Computed tomography
DAB: 3,3′-diaminobenzidin-tetrahydrochlorid
DFS: Disease-free survival
Fig: Figure
Gy: Grey
H&E: hematoxylin–eosin staining
HR: Hazard ratio
HRP: Horseradish-peroxidase
IHC: Immunohistochemical staining
LAR: Low anterior resection
MeV: electronvolt
OS: Overall survival
P: *P*-value
pCR: pathological complete response
SD: Standard deviation
TME: Total mesorectal surgery
TNM: Tumor, node, and metastasis staging system
